# The High Cost of Misdiagnosis: The Challenges of Differentiating Grade-4 Astrocytoma from Glioblastoma in Resource-Constrained Countries

**DOI:** 10.12669/pjms.42.8.16057

**Published:** 2026-08

**Authors:** Hasan Saeed, Haseeb Mehmood Qadri

**Affiliations:** 1 Dr. Hasan Saeed, MBBS. Department of Histopathology, Shifa International Hospital, Islamabad, Pakistan; 2 Dr. Haseeb Mehmood Qadri, MBBS, CME. Department of Neurosurgery, Unit-I, Punjab Institute of Neurosciences, Lahore General Hospital, Lahore, Pakistan

Brain tumors due to their aggressive behavior and challenging sites of origin are one of the most lethal neoplasms. Amongst these, 80-85% are gliomas that diffusely infiltrate the brain parenchyma.^1^ The annual death rate from high grade gliomas being 4.42 per 100,000 with a five-year survival rate of 6.9% is a highlight of their ghastly demeanor.^2^ Glioblastoma is a high grade glioma, which normally affects non-Hispanic Caucasion males in their 70’s has a median survival rate of less than two years. It is the most common primary brain tumor in adults. Low grade gliomas, encompassing Astrocytoma and Oligodendrogliomas, common below the age of 50 have a better survival rate.^1^,^2^

The 5^th^ edition of the World Health Organisation (WHO) Classification of Central Nervous System (CNS) tumors has integrated specific molecular alterations in addition to histology in classifying brain tumors. This has tremendously affected their neoplastic behavior, treatment selection and overall prognosis.^3^ Segregating adult-type diffuse gliomas from pediatric gliomas, incorporation of IDH1/2, ATRX, EGFR and TERT promoter mutational testing are one of the most notable changes in the new edition.^4^ Our focus is solely on CNS WHO Grade-4 Astrocytomas and Glioblastomas. Previously, tumors exhibiting necrosis and microvascular proliferation on histology were classified as Grade-4 Astrocytomas, commonly referred to as Glioblastomas. However, with the recent WHO updates, the presence of specific molecular alterations such as EGFR amplification, TERT promoter mutations, gain of chromosome 7, and loss of chromosome 10 are critical for diagnosing Glioblastoma, IDH wild-type, Grade-4. Conversely, an IDH-mutant Astrocytoma exhibiting these molecular features is classified as Astrocytoma, IDH mutant, Grade-4.^2^,^4^ Ersoz et al. observed no statistically significant difference in the histology of these morphologically similar entities.^5^ Therefore, differentiating these on standard hematoxylin & eosin (H&E) staining is extremely cumbersome. Schaff et al. explained that due to their distinct molecular behavior, these tumors are managed differently. IDH wild-type gliomas require gross total excision, followed by adjuvant radiotherapy usually coupled with an alkylating agent i.e. temozolamaide. Still after extensive therapy, recurrence is inevitable, with a median progression-free survival (PFS) of approximately seven months.^1^ On the other hand, maximum safe resection, followed by addition of procarbazine, lomustine, and vincristine (PCV) to radiotherapy is utilized in IDH-mutant gliomas.^1^ Orsanu et al. explained in their comparative radiological and histological analyses of Glioblastoma IDH wild-type, Grade-4 and Astrocytoma IDH mutant, Grade-4, the lesser evil nature of the latter by assessing their impact on patient survival along with the currently existing therapeutic possibilities.^2^ These unique biological characteristics emphasize the significance of genetic and molecular testing even more.

Issue that needs to be addressed here is what happens to patients diagnosed with Grade-4 Astrocytoma (Glioblastoma) prior to the WHO updates? They might harbor an IDH1/2 mutation, changing their diagnosis to Astrocytoma IDH mutant, Grade-4. Or they might have a TERT promoter mutation, which in any case would alter the diagnosis to Glioblastoma IDH wild-type, Grade-4. Johnson et al. in 2015 described a case diagnosed as a Grade-4 Astrocytoma, that harbored a MGMT methylation.^6^ But according to the recent updates, Grade-4 gliomas harboring this mutation are inevitably classified as Glioblastoma IDH wild-type, Grade-4. The question that might come to everyone’s mind would be why not opting for molecular testing, if histology cannot segregate the two entities. But majority of low and middle income countries (LMICs) cannot afford cytogenetics or molecular testing due to financial restraints. Even if they can afford a molecular biology laboratory, who would run the machines? A proficient and a highly skilled technological staff would be required. Some would say, why not choose immunohistochemistry (IHC) instead? Most of the well-established laboratories usually have the wherewithal for this facility. Singh et al. utilized Anti-IDH1 (R132H) antibody to diagnose and segregate Astrocytoma IDH mutant, Grade-4 from of Glioblastoma IDH wild-type, Grade-4.^7^ But to completely rule out an IDH mutation, both IDH1 and IDH2 genes need to be tested. Unfortunately, no antibody against mutant IDH2 gene is available in the market yet. In the end, the only solution to this query is ‘molecular testing’.

What is the solution? In resource limited countries like Pakistan, one should always go for a second opinion. The tissue blocks should be submitted to an entrenched laboratory, where at least the facility of IHC is available. Preusser et al compared anti-IDH1 IHC and IDH1 gene sequencing and found concordant results in 98.9% (94) cases.^8^ Thus detecting IDH1 mutational status via IHC is authentic. Furthermore, if no mutation is detected in a Grade-4 glioma on IHC, it must not be overtly labeled as Glioblastoma IDH wild-type, Grade-4. Molecular analysis for an IDH2 mutation should be advised in the comments section to confirm the diagnosis in the final histopathology report. Additionally, as previously mentioned, detecting a TERT promoter and/or chromosome 7 and 10 mutation in a glioma of any grade renders a diagnosis of Glioblastoma IDH wild-type, Grade-4. A comment should be made in this regard as well.

To conclude, diagnosing CNS pathology has come a long way. Everything in the present relies on the molecular genetics and genome biology. New mutant genes are being discovered with every passing day. This is going to have a significant impact on the CNS tumor classification and in turn their therapeutic management. Dealing with these enigmatic tumors is a constant battle for both clinicians and histopathologists. The best thing we can do is to make a safe and up to par diagnosis, addressing all the limitations in the final report.

## Authors contributions:

**HS** is responsible for data acquisition, analysis and drafting the manuscript.

**HMQ** is responsible for concept, design of the study and critically reviewing the manuscript.

Both authors agree to the final version of the manuscript and agree to be accountable for all aspects of its integrity after publication.
